# Frameworks for targeting RNA with small molecules

**DOI:** 10.1074/jbc.REV120.015203

**Published:** 2020-12-20

**Authors:** Aline Umuhire Juru, Amanda E. Hargrove

**Affiliations:** Department of Chemistry, Duke University, Durham, North Carolina, USA

**Keywords:** RNA-targeted therapies, RNA folding, RNA structure, small molecule, drug discovery, long noncoding RNA, miRNA, riboswitch, trinucleotide repeat disease, ASO, antisense oligonucleotide, DM1, myotonic dystrophy type 1, lncRNA, long noncoding RNA, R-BIND, RNA-targeted BIoactive ligaNd Database

## Abstract

Since the characterization of mRNA in 1961, our understanding of the roles of RNA molecules has significantly grown. Beyond serving as a link between DNA and proteins, RNA molecules play direct effector roles by binding to various ligands, including proteins, DNA, other RNAs, and metabolites. Through these interactions, RNAs mediate cellular processes such as the regulation of gene transcription and the enhancement or inhibition of protein activity. As a result, the misregulation of RNA molecules is often associated with disease phenotypes, and RNA molecules have been increasingly recognized as potential targets for drug development efforts, which in the past had focused primarily on proteins. Although both small molecule–based and oligonucleotide-based therapies have been pursued in efforts to target RNA, small-molecule modalities are often favored owing to several advantages including greater oral bioavailability. In this review, we discuss three general frameworks (sets of premises and hypotheses) that, in our view, have so far dominated the discovery of small-molecule ligands for RNA. We highlight the unique merits of each framework as well as the pitfalls associated with exclusive focus of ligand discovery efforts within only one framework. Finally, we propose that RNA ligand discovery can benefit from using progress made within these three frameworks to move toward a paradigm that formulates RNA-targeting questions at the level of RNA structural subclasses.

In 1947, Boivin and Vendrely (1) hypothesized that DNA produced RNAs that then produced different proteins. In the 14 years that followed, the characterization and isolation of mRNA were reported by two research groups—Brenner, Jacob, and Meselson on the one hand, and Gros *et al*. on the other—in 1961 (2, 3, 4). This same year, Nirenberg and Matthaei demonstrated the function of mRNA *via* an array of *in vitro* translation experiments, one of which showed that a poly-U RNA molecule acting as a template resulted in polyphenylalanine peptide (2). These years proved a critical period in the history of the genetic code. Prior to these discoveries, although DNA was accepted as the locus of genetic information, it was still obscure just how this information was transferred to proteins. The central dogma of molecular biology, which Francis Crick had proposed in 1957, was thus fully established.

From 1961 onward, apart from rRNA and tRNA discovered in 1955 and 1957, respectively, RNA molecules were largely viewed simply as messengers. This view began to change with the discovery of catalytic RNAs by Thomas Cech and Sydney Altman in the early 1980s as well as the discovery of regulatory RNAs that did not code for proteins (5). The earliest class of regulatory ncRNA discovered consisted of small RNAs that regulate mRNA translation in prokaryotes (5). The discovery of miRNA in the early 1990s through observations by multiple researchers continued to lend evidence to the fact that RNA could directly perform regulatory function (5). The early 1990s also marked the discovery of the first long ncRNA (lncRNA), H19, important in mammalian embryonic development, as well as lncRNA X-inactive-specific transcript, which is responsible for X-chromosome inactivation in mammalian females (XX) to achieve dosage compensation relative to males (XY) (5). These early discoveries of ncRNAs, along with the observation that most of the human genome produces noncoding transcripts (5, 6), led to the so-called revolution in RNA biology, a shift toward an increased appreciation of RNA functions beyond templating protein synthesis.

Today, several classes of ncRNAs with diverse functions have been identified, and their structures and functions are being further investigated. These include miRNAs mentioned previously that regulate protein expression (7), small nucleolar RNAs involved in ribosomal RNA modification and mRNA splicing (8, 9), small nuclear RNAs involved in splicing (10), lncRNAs (operationally defined as RNAs with >200 nucleotides) (11), and many more (12). LncRNAs, in particular, are involved in several cellular processes, including the regulation of chromatin architecture, transcriptional regulation, inhibition or enhancement of protein activity (5), and the regulation of nuclear bodies (13). In addition, lncRNAs have been implicated in the progression of various cancers. For example, lncRNA metastatic-associated lung adenocarcinoma transcript 1 is overexpressed in many cancers and associated with tumor growth and metastasis (14). Noncoding portions of mRNAs can also be implicated in disease progression, as is the case with many neurological disorders that result from the expansion of trinucleotide repeats in untranslated regions of a key mRNA (15).

With the discovery of functional RNA molecules, it became clear that drug discovery, which had previously focused solely on proteins, should also be applied to RNA. This approach carries great potential for at least three reasons. First, targeting a misregulated disease-related RNA might be more amenable to drug development especially if targeting proteins involved in the same pathway may lead to undesirable side effects. For example, achieving selectivity when targeting structurally related proteins such as kinases may prove difficult, while targeting the respective mRNAs may allow a higher level of selectivity (16). Second, for some proteins currently considered difficult to drug, it might only be possible to modulate their effect by targeting the corresponding mRNA (17). Finally, several functional RNAs have been found to play essential roles in the proliferation of viral, fungal, and bacterial pathogenic organisms (18, 19, 20). For example, the early 2000s marked the discovery that some mRNAs could regulate their own expression either at the transcriptional or at the translational level by directly binding to metabolites without involvement of a protein sensor (21, 22, 23, 24, 25). The RNA structural elements responsible for this regulation, termed riboswitches, have so far been identified in all three domains of life (26). In many cases, RNAs essential for pathogenic organisms do not have close orthologs in humans, which makes them orthogonal targets, thus increasing the chances of selective targeting. Targeting RNA thus opens the door to novel treatments for both infectious and noninfectious diseases.

Early interest in modulating the function of RNA dates back to 1978 with the study of an oligonucleotide inhibiting replication of Rous sarcoma virus (27). Possible mechanisms included blocking translation initiation. In 1998, the US Food and Drug Administration (FDA) approved the antisense oligonucleotide (ASO) Vitravene for cytomegalovirus retinitis, although it has now been discontinued (16). Vitravene inhibited the synthesis of proteins essential for viral replication by binding to the mRNA sequence encoding these proteins (28). Another ASO, Kynamro (mipomersen), was approved by the FDA in 2013 for familial hypercholesterolemia (16). Interaction of Kynamro with apolipoprotein B-100 mRNA induced its cleavage by ribonuclease H, leading to reduced production of lipoproteins (29, 30). More recently, Spinraza (nusinersen), an ASO targeting pre-mRNA splicing, was also approved by the FDA for spinal muscular atrophy. Nusinersen bound to an intron-silencing sequence in the *SMN2* pre-mRNA and induced the inclusion of exon 7 resulting in production of the full-length survival motor neuron protein (31). Other protein replacement approaches have included the delivery of mRNA therapeutics as replacement for deficient endogenous sequences (32). These initial successes with ASOs would find even more appreciation as their applicable domain expanded with the discovery of ncRNAs. Similar to how they block mRNA translation either sterically or by inducing cleavage, ASOs can bind to ncRNAs and induce their cleavage or block their interactions with endogenous ligands (*e.g.*, proteins, nucleic acids, metabolites, etc), ultimately abrogating downstream cellular processes. Additional RNA-cleavage mechanisms not mediated by ASOs have also been pursued. These include the development of siRNAs, which are double-stranded RNAs that recognize and induce cleavage of a target RNA *via* endogenous RNAi pathways. The first RNAi therapy, Patisiran, was approved in 2018 for hereditary transthyretin amyloidosis, an autosomal dominant neurodegenerative disease (33). The siRNA Patisiran binds in the 3ʹ untranslated region of mutant and wildtype transthyretin mRNA, inducing cleavage of mRNAs and thus reducing the deposition of transthyretin proteins (34, 35).

Although the FDA approval of the siRNA Patisiran represented significant progress, the nearly two-decade delay between the discovery of RNAi in the early 2000s (36, 37) and the first approval of an RNAi-based therapy points to challenges inherent to developing oligonucleotide-based therapies that limit widespread use compared with traditional small-molecule drugs (33, 38). For example, although small single-stranded ASOs can be taken up by cells and escape the endosome more readily than larger agents like double-stranded siRNAs, their delivery to nonhepatic tissues is still difficult. In particular, given that ASOs cannot cross the blood–brain barrier, their application to treat neurological diseases requires direct injection into the spinal canal (38). In addition, oligonucleotide therapies can elicit both extracellular and intracellular immunological responses. Small organic molecules, on the other hand, can be orally bioavailable with systemic delivery and are not immunogenic. Finally, given that oligonucleotides work through base pairing, it proves difficult to target structured RNAs without accompanying structural rearrangement. In contrast, the high tunability of small-molecule physiochemical and shape properties allows them to target diverse highly structured RNA motifs. These advantages of small molecules, along with the desire to apply the cumulative knowledge in medicinal chemistry to these newly appreciated RNA targets, have turned the interest of targeting RNA from oligonucleotides to traditional small molecules. This interest is particularly exemplified by the growing number of academic research laboratories and startup companies that are dedicating their efforts to developing small molecules that directly bind RNA to alter function.

The earliest class of small molecules known to interact with RNA and modulate RNA function was identified in the late 1980s when it was discovered that aminoglycoside antibiotics acted by binding to bacterial ribosomal RNA (39). Researchers soon observed that these molecules could bind to a variety of nonribosomal RNAs, mainly because of the high content in positively charged amino groups that lend a certain degree of nonspecific binding to RNA because of its negatively charged backbone. Several researchers viewed this promiscuity as a potential opportunity and focused on tuning aminoglycoside derivatives for various RNA targets. As RNA was increasingly viewed as a potential drug target, research efforts were expanded to also include more general drug-like small molecules often described as those with physiochemical properties satisfying Lipinski's “rule of 5” (40, 41), although other methods to describe drug likeness have been proposed (42).

In this review, we will discuss the main research frameworks that have driven the study of targeting RNA with small molecules. We define a framework as a set of premises and hypotheses underlying any individual research strategy. For example, a study of how the properties of small molecules affect their interaction with differentially sized RNA bulges would be operating within a framework that considers RNA secondary structures to play an important role in determining ligand interaction. In our evaluation of RNA-small molecule literature, we observed that many of the approaches to RNA ligand discovery have so far operated within three main frameworks: (1) RNA secondary structure motifs can be used as modules for ligand binding; (2) RNA-targeted small molecules may have distinct properties compared with protein-targeted molecules; and (3) RNA-targeted small molecules may look like typical drugs targeting proteins. For each framework, we will first discuss how it is generally conceived and implemented and then we will provide a critical evaluation in relation to other frameworks. Having focused our analysis mainly on major themes, we will not discuss important work that may not fit into the three main frameworks such as modulation of the RNA conformational landscape, which we discussed in a previous review article (43), or the use of small molecules to induce degradation of pathogenic RNAs (44).

## Framework 1: RNA secondary structure motifs can be used as modules for ligand binding

**Figure 1:**
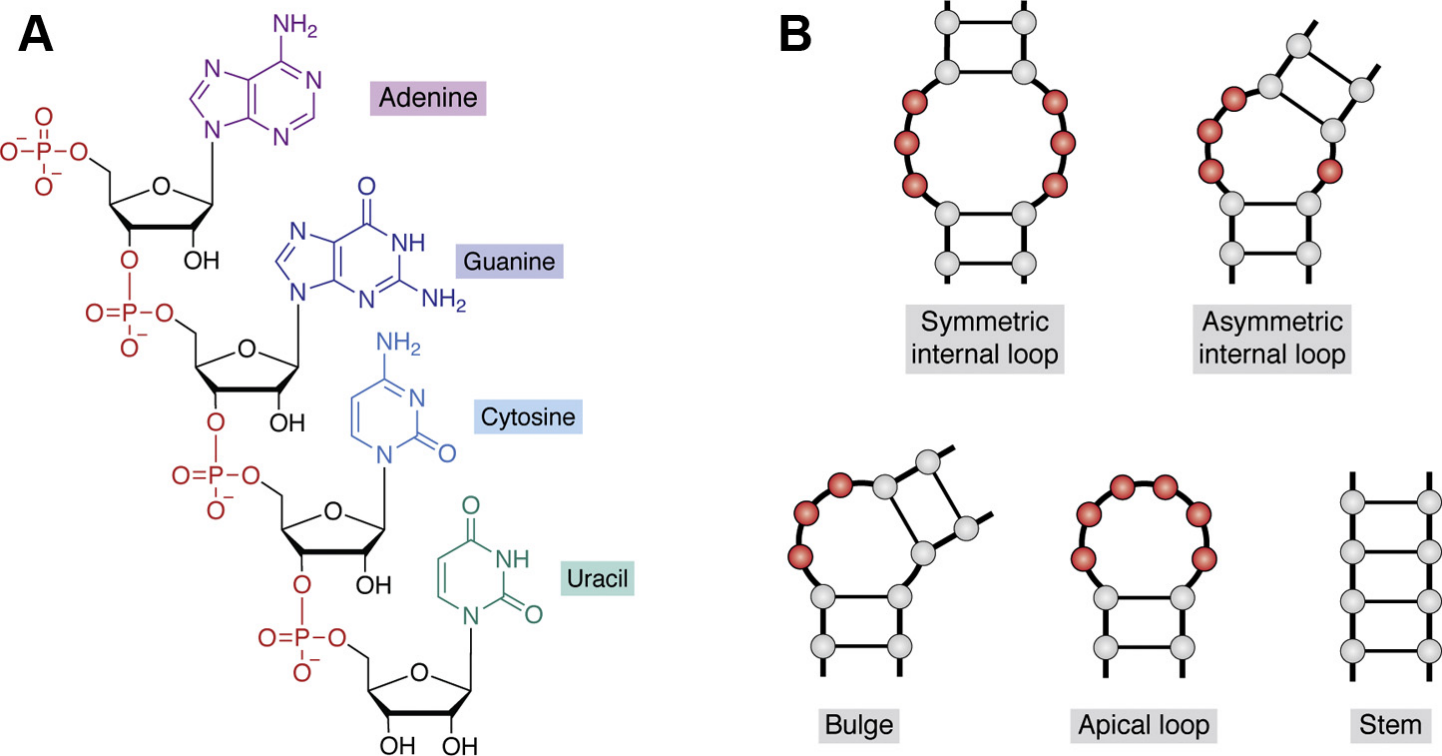
**Chemical composition and secondary structure motifs of RNA.***A*, chemical structure of a sample RNA strand composed of the four bases found in RNA. Because they utilize only four monomers, RNA molecules are often considered to have low chemical diversity compared to proteins that are made of 22 amino acids (46). In addition, unlike proteins that exhibit a wide range of net charge, RNA molecules are negatively charged at physiological pH because of the acidic phosphate backbone. *B*, canonical secondary structure motifs of RNA. RNA molecules fold through complementary base pairing. In addition to the canonical A–U and G–C base pairs, RNA folding also utilizes non-Watson–Crick base pairs such as the well-studied G–U wobble pair (47) and several others (48). Unpaired regions are highlighted in *red*.

Early investigations in how aminoglycosides interact with RNA showed that recognition may depend on RNA shape and not on sequence (45). In the absence of complex tertiary interactions as is the case for the short stem loops often used in *in vitro* experiments, the shape of a potential binding site is defined by the secondary structure motifs (*e.g.*, loop, bulge, etc; Fig. 1*B*). The uniqueness of this shape originates from the size of the unpaired region and the identity of the unpaired bases and their neighbors. Therefore, to target an RNA with small molecules, it might be beneficial to focus on the unique set of secondary structure motifs it contains. This idea quickly matured into a more general approach to solving the problem of RNA recognition with small molecules.

For example, the Hergenrother laboratory (49, 50, 51) published several studies aimed at targeting apical loops or bulges selectively. In a study aimed at identifying general RNA apical loop binders, the Hergenrother laboratory (49) reported deoxystreptamine dimers that had high affinity for apical loops. In a follow-up study, a combinatorial library of deoxystreptamine dimers was synthesized and evaluated for size-specific binding of RNA apical loops (50). Compounds with selectivity for octaloops and others with selectivity for tetraloops were identified. In addition to apical loops, the Hergenrother laboratory also investigated bulge-binding compounds. In one study, they synthesized a library of compounds with a wedge-like geometry that gives them high affinity for nucleic acid bulges. In this compound series, the wedge-like geometry was essential for binding while cationic character was not as important (51).

**Figure 2:**
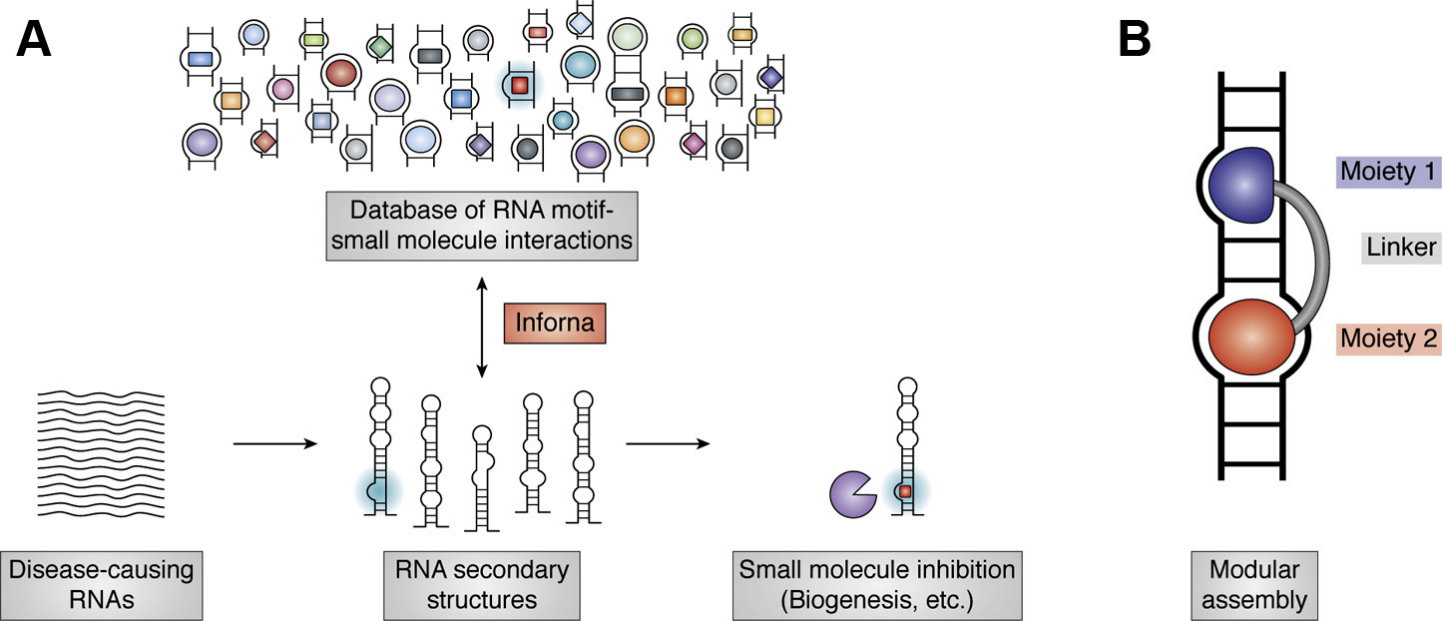
**Example study in Framework 1**. *A*, illustration of how the Inforna database is used to identify small molecules interacting with a disease-causing RNA of interest. Inforna compares secondary structure motifs in the target of interest with those found in the database and then outputs small molecules predicted to bind to one or more of the secondary structure motifs in the target RNA. The figure was adapted from Disney and Angelbello (56) with permission. Copyright (2016) American Chemical Society. *B*, the modular assembly technique where moieties binding neighboring secondary structure motifs are linked together to increase potency.

The Disney laboratory took a large-scale approach to identify what secondary structure motifs are preferred by what molecules (52, 53, 54, 55). In a seminal study, Disney and Childs-Disney (52) screened a randomized library of internal loops against a kanamycin A derivative to identify what types of internal loops, in terms of size and sequence, kanamycin A prefers to bind. They proposed using this type of information to construct a database of RNA motifs that small molecules recognize. This database could then be used to identify small molecules that can be linked to bind sequential secondary structure motifs in an RNA of interest (Fig. 2). Using a technique termed two-dimensional combinatorial screening, the Disney laboratory constructed the proposed database, now known as Inforna, which they have used to target several RNAs with small molecules possessing activity in cell culture and/or animal models of cancer and neurological disorders (53, 54, 55, 57, 58, 59, 60). A key strategy in Disney's work has been that of modular assembly, where at least two binding moieties are linked together to interact with neighboring secondary structure motifs such as those found in precursor miRNAs and trinucleotide repeat RNAs (Fig. 2*B*).

A similar strategy using different building blocks has also been used by the Zimmerman group to target trinucleotide-repeat RNAs with ligands active in cell culture and animal models (61, 62, 63, 64). In an effort to target the CUG repeat RNA in myotonic dystrophy type 1 (DM1), the Zimmerman group developed multivalent ligands consisting of two acridine–triaminotriazine moieties connected by an oligoamino or oligoether linker (61). Given the multivalent nature of the target RNA (repeating U:U internal loops), utilization of dimeric compounds with an optimized linker was expected to improve potency. One dimeric compound in this series was found to have over 200-fold greater potency than the monovalent parent ligand in *in vitro* assays. In a recent study, the multivalent approach was further expanded to produce oligomeric compounds composed of alternating bisamidinium and triaminotriazine moieties (64). These compounds were designed to bind both the CUG repeat RNA as well as the corresponding CTG repeat DNA to provide a multitargeting approach to developing treatments of DM1. Importantly, in addition to improving nucleic acid binding, the presence of multiple bisamidinium moieties in these oligomeric compounds also served to improve cellular uptake of the compounds, presumably through the mechanisms employed by cell-penetrating peptides (64). As a result, the final compound showed greater efficacy in cellular and *in vivo* assays compared with the monomeric compound.

The idea that secondary structure motifs can serve as modules, or units, for targeting RNA has delivered several bioactive small molecules (53, 54, 55, 57, 58, 59, 60, 61, 62, 63, 64). These successes testify to the strength of this approach as a general RNA-targeting strategy. However, this approach remains limited in its application, as it seems applicable only to simple stem-loop structures such as miRNA precursors and trinucleotide repeat RNAs and is not as easily applied to complex three-dimensional pockets such as those found in higher-level junctions. In addition, because highly similar secondary structure motifs can be found in multiple RNAs, targeting one RNA selectively using Framework 1 may prove difficult. As a result, this approach may be limited to targeting overexpressed RNA transcripts, as this abundance can significantly contribute to small-molecule selectivity even in the presence of RNAs that may have similar secondary structure motifs.

## Framework 2: potential existence of an RNA-biased chemical space

Similar to Framework 1, the second dominant framework in RNA ligand discovery is also based on the dissimilarity of RNA as a biopolymer compared with proteins. Unlike Framework 1, however, Framework 2 is not focused on modules of RNA structure that can be used as units in designing selective small molecules. In this framework, researchers take the RNA molecule as a whole, and after considering its unique properties—such as its high negative charge and relatively low chemical diversity (Fig. 1*A*)—they hypothesize that, in general, RNA-binding small molecules will have distinct structural properties compared with protein-binding small molecules. As such, there may exist a region of chemical space that is privileged or biased to interact with RNA (65). Given that compound libraries compiled by medicinal chemists before the broad acceptance of RNA as a drug target were mostly aimed at targeting deep hydrophobic pockets of proteins (66), it follows that the research focus turns to identifying new types of small-molecule scaffolds that interact with RNA and to designing compound libraries that have been statistically biased to yield a higher number of hits for RNA targets.

### Scaffold-based synthesis

The first main approach within Framework 2 has been scaffold-based synthesis, where a molecular scaffold known to interact with an RNA molecule is further diversified to produce analogs that are optimized for differentially modulating different RNA structures. The molecular scaffold retains core structural features that confer general RNA binding, whereas the substitution pattern allows the researcher to engineer selectivity for a desired RNA molecule. In some cases, scaffold-based research programs originated from the realization that DNA-interacting molecules could potentially be tuned to interact with RNA. Such was the case with some of the work from the Wilson and Zimmerman laboratories where expertise in targeting DNA with aromatic heterocyclic compounds and molecular tweezer–like compounds, respectively, was applied to RNA targets (67, 68). The Miller laboratory also used a similar approach. A dynamic combinatorial library initially designed for DNA using building blocks from DNA-interacting natural products was later successfully employed for RNA targets (69, 70).

In addition to DNA-inspired research efforts, already-known RNA-binding scaffolds such as the aminoglycosides were further investigated in an effort to fine-tune them for specificity. Several laboratories have published extensively on aminoglycosides (71, 72, 73, 74, 75, 76, 77, 78, 79, 80, 81, 82, 83, 84, 85, 86), including some of the early efforts to target RNA in a multivalent manner (82) as well as the first examples to reveal the importance of structural electrostatic complementarity between an RNA target and a small molecule (81). A general theme emerging from the aminoglycoside work has been the difficulty of achieving selectivity between different RNAs—although different strategies were pursued including conformational restriction and conjugation of aminoglycosides to other moieties, achieving selectivity remained difficult (75, 83, 85). It is noteworthy, however, that in some instances, the lack of selectivity appeared to be coming from the flexibility of the RNA target rather than inherent promiscuity of the compounds (75, 85).

**Figure 3:**
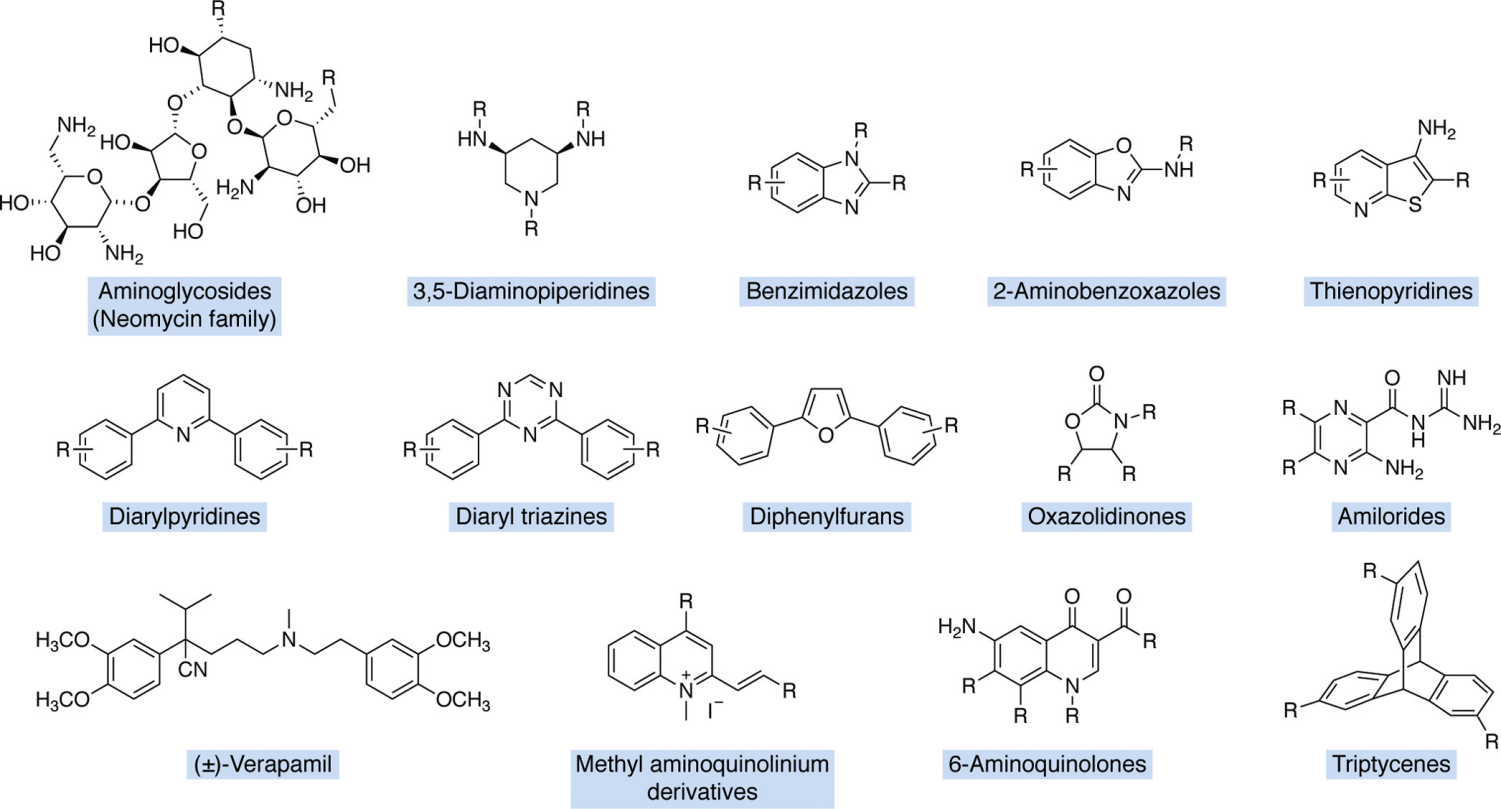
**Example of small-molecule classes that have been pursued through scaffold-based synthesis**. R groups represent substituents used to diversify the central core scaffold.

Finally, high-throughput screening approaches have led to identification of novel highly tunable scaffolds, as is the case with the amiloride scaffold initially identified by the Al-Hashimi laboratory (87). Our laboratory has since synthesized ∼60 derivatives, some of which have high specificity for select viral RNAs (88, 89, 90). Other scaffolds investigated for RNA binding through scaffold optimization have included benzimidazoles (91), aminoglycoside–benzimidazole conjugates (92, 93, 94, 95), 2-aminobenzoxazoles (96), thienopyridines (97), diarylpyridines (98), diaryltriazines (99), oxazolidinones (100), 3,5-diamino-piperidines (101, 102), diphenylfurans (14, 67, 103, 104, 105), verapamil (106), methylquinolinium derivatives (107), aminoquinolones (108), and triptycene-based molecules designed for DNA and RNA junctions (109) (Fig. 3).

### RNA-biased libraries

The second major approach in Framework 2 has focused on studying structural properties of RNA-binding molecules and using these properties to design RNA-biased libraries. For example, the Disney laboratory designed a library enriched in moieties observed to interact with RNA in their earlier two-dimensional combinatorial screening studies (110). This library was screened against the r(CUG)^exp^ RNA in myotonic dystrophy and resulted in a higher hit rate than typically observed for general high-throughput libraries. From an efficiency standpoint, higher hit rates in RNA screens are often desired especially in academic settings where the libraries available are orders of magnitude smaller than those available to large companies. As such, creating RNA-biased libraries may be highly advantageous in RNA lead discovery, especially because compound libraries have so far been optimized around protein binding (66). Bodoor *et al*. (111) used an analogous approach, where published RNA ligands were fragmented, and an RNA-biased library was assembled based on structural similarity to the original RNA-binding fragments. This library led to the identification of five hits for the bacterial ribosomal A-site RNA, including chemotypes not previously known to interact with this RNA. However, this work did not allow general conclusions about small-molecule properties that lead to RNA binding.

**Figure 4:**
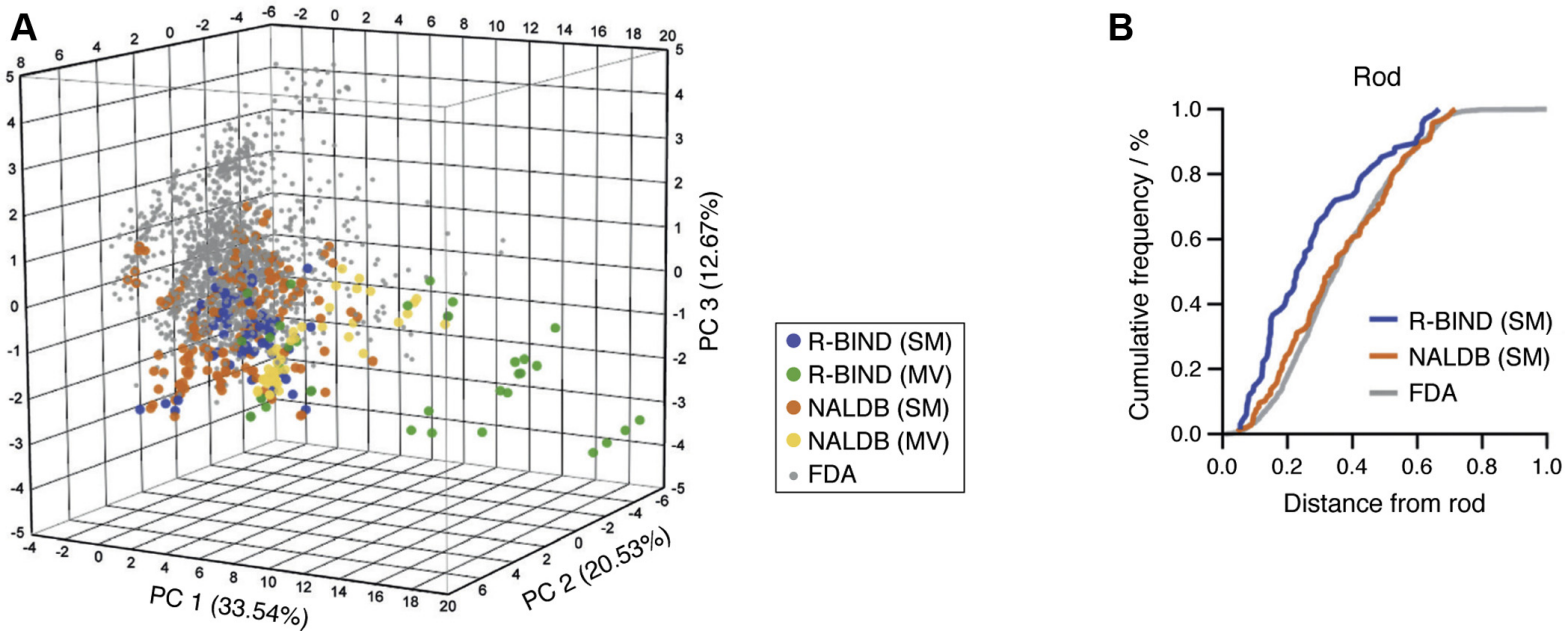
**Comparison of molecules in the RNA-targeted BIoactive ligaNd Database (R-BIND) to Food and Drug Administration (FDA)–approved drugs and general nucleic acid–binding ligands (NALDBs)**. *A*, principal component analysis on 20 calculated cheminformatics parameters. R-BIND ligands occupy a focused region of the chemical space defined by the three libraries. *B*, ligand shape expressed in terms of rod likeness. The R-BIND database is enriched in molecules with rod-like character compared with the FDA and NALDB libraries. SM denotes the monovalent small-molecule category within R-BIND and NALDB, whereas MV denotes multivalent compounds defined as having two binding moieties connected by a linker and a molecular weight greater than 500 amu. The figure was adapted from Morgan *et al*. (112) with permission. Copyright (2017) Wiley-VCH Verlag GmbH & Co KGaA, Weinheim.

Our laboratory recently took a complementary approach to investigating RNA-privileged chemical space (112, 113). The approach was heuristic similarly to the examples discussed previously, but we focused on ligands that had biological activity in cells or animal models. Bioactive compounds were expected to already possess some level of selectivity because they recognize their target RNA in a cellular context, where ribosomal and tRNA make up over 90% of total cellular RNA (114). Analysis of published RNA-targeted bioactive ligands showed that these compounds have unique trends in structural and shape properties compared with FDA-approved drugs, which are considered to mostly target proteins, while at the same time having similar medicinal chemistry properties (112) (Fig. 4). For example, compounds in the Hargrove RNA-targeted BIoactive ligaNd Database (R-BIND) had a higher nitrogen count, a higher number of aromatic rings, a lower oxygen count, a lower fraction of sp^3^-hybridized carbon atoms, and a lower number of stereocenters. R-BIND compounds also had a more rod-like shape compared with FDA-approved drugs. This work suggested that bioactive RNA-targeted ligands may occupy a focused corner of drug-like chemical space. These results were further supported by recent work from the Disney laboratory in collaboration with AstraZeneca (57). The researchers observed that although the RNA-binding hit compounds were structurally dissimilar to those in R-BIND (113), they still shared the identified physicochemical properties. We envision that these physicochemical properties could be harnessed by designing an RNA-biased library using compound similarity algorithms on R-BIND ligands, potentially allowing the identification of novel ligands for a variety of RNA targets.

Rizvi *et al*. (115) recently published a study in which they investigated small-molecule properties that lead to selective RNA binding. They screened a library of ∼50,000 drug-like and 5100 tool compounds against a variety of RNA structures using a mass spectrometry-based assay and used a machine-learning algorithm on identified binders to build an RNA-biased library, which had a higher hit rate compared with the starting libraries. Similar to the studies discussed previously, Rizvi *et al*. (115) observed that RNA-binding ligands were found within drug-like chemical space but that there were chemical substructures, particularly heteroaromatic rings, that promoted general RNA binding.

The observation that RNA-binding ligands may have distinct features compared with general protein binders yielded several lines of research, which we discussed under two umbrella approaches—scaffold-based synthesis and the design of RNA-biased libraries. Both these approaches have advanced the field of targeting RNA through the identification and optimization of new scaffolds as well as through the higher hit rates obtained with biased libraries that expedite lead discovery. For example, one of the first ligands for a lncRNA structural element was identified in our laboratory *via* diversification of a diphenyl furan scaffold (14). Screening of RNA-biased libraries has also led to successful targeting of RNA in disease, as demonstrated by the Disney study that was enriched in RNA-binding features, including the benzimidazole moiety, and led to compounds that improve splicing defects in DM1 (110). Importantly, we note that in contrast to Framework 1, which is limited to simple stem loops, Framework 2 has allowed the targeting of complex structures such as an lncRNA triple helix structure (14), G-quadruplexes (107), and riboswitches (116, 117), thus establishing it as a general RNA-targeting strategy.

## Framework 3: RNA-targeted small molecules may look like typical drugs targeting proteins

**Figure 5:**
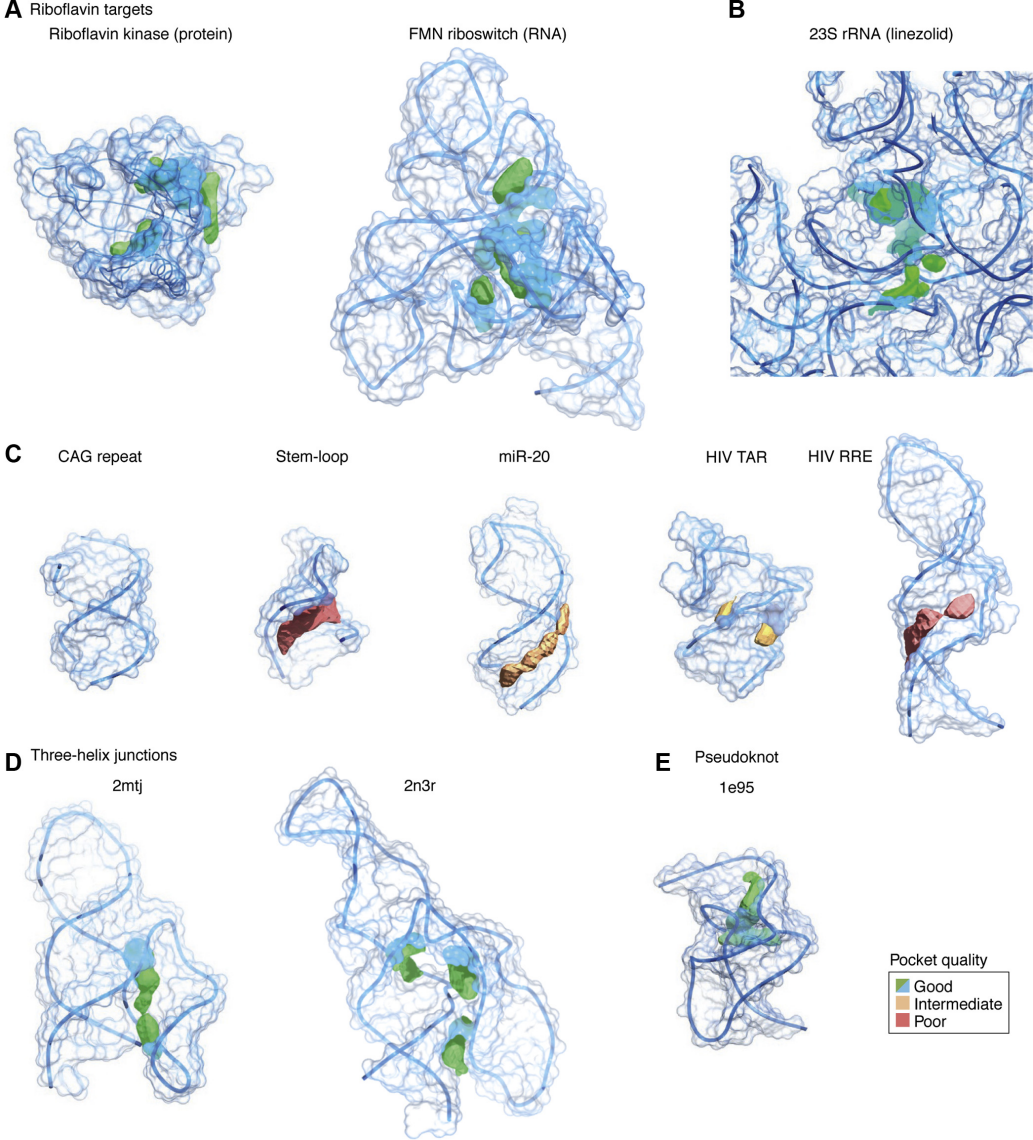
**Pocket analysis in RNA structures performed by Warner *et al***. (17). Complex structures have good quality pockets (*green*/*blue*), whereas stem loops have poorer pockets (*orange* and *red*). *A* and *B*, currently targeted RNA structures with good quality pockets. *C*, currently targeted RNA structures with low-to-medium quality pockets. *D* and *E*, aspirational targets with potential good quality pockets. The figure was reprinted with permission from Springer Nature Customer Service Centre GmbH: Springer Nature, Nature Reviews Drug Discovery (17), Copyright 2018.

From our discussions of RNA-biased chemical space in Framework 2, we observe that RNA-binding small molecules often have distinct properties compared with general protein-targeted drugs. However, the two groups are often found within the same larger chemical space, sharing properties that include those used to define drug likeness such as Lipinski's and Veber's rules (112). It is this key concept of similarity that expands and becomes the foundation for Framework 3, which posits that traditional medicinal chemistry approaches can readily be applied to RNA targets. The key defining feature of Framework 3 is that researchers can take their focus away from the uniqueness of RNA and redirect it to its similarity to proteins. For example, recent discourse in the field points to the opportunity of targeting protein-like binding pockets in higher level folding RNA structures. This perspective was recently discussed in great detail by Warner *et al*. (17) in their 2019 perspective article. They emphasized the importance of choosing RNAs with complex structures (Fig. 5) in order to achieve both potency and selectivity and argued that, in this way, targeting RNA would become (only) roughly as difficult as for protein targets. While this approach may increase the overall number of RNA molecules targeted with small molecules, we note that an exclusive focus on complex structures may preclude opportunities to develop needed medicines for conditions mediated by RNAs with simpler structures.

Although Framework 3 is not yet as established in the literature as Frameworks 1 and 2, a few recent studies applied this concept. For example, Fedorova *et al*. (18) recently reported a study targeting fungal group II self-splicing introns in which they identified novel antifungal agents *via* a standard drug discovery approach used in pharmaceutical companies—high-throughput screening for inhibitory activity, followed by structure–activity relationship studies for lead optimization. In this report, the authors highlight the approach taken as a significant contribution to efforts toward targeting RNA. Fedorova *et al*. (18) pointed out that although previous studies focusing on identifying physicochemical properties that lead to selective RNA recognition have been successful, they remain limited because they rely on already-known RNA-small-molecule interactions. With the successful application of a standard drug discovery approach to an RNA target, the authors concluded that determinants for RNA binding are sufficiently similar to those for protein targeting to warrant use of established medicinal chemistry libraries, and that targeting complex RNA molecules will not necessarily require a reimagined RNA-centric medicinal chemistry approach.

Another example of applying a traditional drug discovery approach to RNA targets was employed by Merck Research Laboratories in their discovery of the antibacterial ribocil (118). Ribocil inhibits the biosynthesis of the essential vitamin B2 (riboflavin) by binding to the FMN riboswitch, which controls the expression of the *rib* biosynthetic genes. The discovery of ribocil occurred *via* a screen of 57,000 antibacterial small molecules for growth inhibition of an antibiotic-sensitized *Escherichia coli* strain in the presence or in the absence of riboflavin (118, 119). Ribocil emerged as the only compound whose effect was fully suppressed by riboflavin supplementation, indicating that it was inhibiting growth through the riboflavin pathway. Ribocil-resistant *E. coli* mutants were then found to have mutations in the FMN riboswitch rather than in the open reading frame of a riboflavin biosynthetic gene, supporting that ribocil binds to the FMN riboswitch to inhibit gene expression. This mode of action was further supported by additional assays including the inhibition of FMN-controlled gene expression by ribocil in a reporter system as well as *in vitro* binding of ribocil to purified FMN riboswitch RNA. The discovery of ribocil and characterization of its mode of action in this manner emphasized that successful targeting of RNAs with small-molecule drugs can be achieved using strategies that have been traditionally used for protein targets (118).

In addition to the ability of medicinal chemists to apply to RNA the libraries and methods they customarily use for protein targets, another important motivation within Framework 3 relates to the danger of relying on already-known RNA-binding ligands, a theme that is particularly salient in Framework 2. As beneficial as it is to utilize properties of known ligands to design new ones, it is unlikely that this approach will work for any RNA target given that the ligands we currently have target only a small subset of the RNA structure space. In addition, researchers may encounter increased challenges with selectivity if the same set of small-molecule scaffolds is continually employed for ligand design. As such, it becomes necessary to balance these efforts with screening approaches that allow expanded exploration of chemical space and are thus likely to yield novel molecules. The Schneekloth, Garner, Campos-Olivas, and González laboratories (120, 121, 122) have highlighted this point in their recent high-throughput screening studies.

Finally, as RNA targeting becomes increasingly pursued using the same strategies and compound collections used to target proteins, the crossfertilization of knowledge from both the RNA-targeting and protein-targeting efforts will benefit both fields. At the level of compound optimization for general features such as drug transport, lessons learned from years of optimizing compounds for cellular uptake can equally benefit RNA-targeted compounds. For example, consideration of compound physicochemical properties that affect passive transport such as the water–octanol partition coefficient (123) will be important in optimizing compounds for targeting RNAs. It is important to note, however, that the relative contribution of passive transport and carrier-mediated transport in drug internalization is a subject of ongoing investigations, with some studies pointing to the coexistence of both mechanisms (123, 124). In addition to gains in compound optimization for transport, the RNA-targeting and protein-targeting fields will benefit at the level of validating the mode of action of lead compounds in that researchers can no longer focus only on one class of biomolecules for assessing binding and functional selectivity. For example, recent publications have emphasized the importance of including RNA molecules in off-target screens of protein-targeted compounds (39). Similarly, researchers developing RNA-targeted compounds should remain mindful of the possibility of off-target interactions with proteins. A recent evaluation of a subset of RNA-targeted ligands with biological activity found that appropriate selectivity assays are often not performed comprehensively, with some compounds exhibiting interactions with assay reporter proteins (125). While it remains difficult for a compound to interact with only one biomolecule in the complex cellular environment, expansion of binding and functional selectivity evaluations both for RNA-targeted and protein-targeted compounds will be crucial to ensure a higher success rate of drug discovery efforts.

## Conclusions and perspectives

With the discovery of myriad functional ncRNAs across all domains of life, many of which lead to disease when misregulated, there has been increased interest in pursuing RNA molecules as drug targets. In our discussion of the three main frameworks that have driven research in this field, we have highlighted the successes and limitations of the versatile approaches taken to target RNA molecules. Importantly, it would appear that one framework may be better suited for certain RNA structure subclasses. For example, Framework 1 may be the best option for targeting a well-characterized functional stem loop, whereas Framework 3 may be better suited for complex structures such as riboswitches. We note that it is possible for a study to fall under two frameworks, as would be the case for a modular approach (Framework 1) that utilizes RNA-biased libraries or scaffold-based synthesis (Framework 2) as the discovery method.

Finally, we would like to discuss an aspect of RNA ligand research that, in our view, underlies all three frameworks discussed herein. This aspect is the tendency to treat RNA as a monolithic collection of biomolecules in the context of medicinal chemistry, and thus requiring (or not) umbrella RNA-centric methods, even as one simultaneously recognizes the structural diversity of RNA molecules in other contexts. This aspect of our current discourse leads to questions like what kinds of molecules can target RNA?, as opposed to what kinds of molecules target triple helices/pseudoknots? or what kinds of molecules target this particular cancer-related RNA fragment? While our attempts to find generalizable approaches at the level of RNA have led to important successes and remain important, it is likely that generalization may be more meaningful and more useful if tailored to a particular structural class of RNA, as routinely done for protein targets. We believe that efforts to target RNA will greatly benefit from approaches that embrace and capitalize on both the similarities between RNA molecules and their rich three-dimensional structure diversity but without being hindered by the former. Importantly, the strategies developed within the three frameworks have laid the groundwork for more detailed exploration of RNA-targeting with small molecules. As such, the field is poised to make significant progress that in time will lead to the discovery of several RNA-targeted life-saving medicines as exemplified by the recent FDA approval of Risdiplam for spinal muscular atrophy (126).

## Conflict of interest

The authors declare that they have no conflicts of interest with the contents of this article.
